# BCOR Internal Tandem Duplication Expression in Neural Stem Cells Promotes Growth, Invasion, and Expression of PRC2 Targets

**DOI:** 10.3390/ijms22083913

**Published:** 2021-04-10

**Authors:** Satoshi Nakata, Ming Yuan, Jeffrey A. Rubens, Ulf D. Kahlert, Jarek Maciaczyk, Eric H. Raabe, Charles G. Eberhart

**Affiliations:** 1 Department of Pathology, Johns Hopkins University School of Medicine, Baltimore, MD 21205, USA; snakata1@jhmi.edu (S.N.); myuan3@jhmi.edu (M.Y.); 2 Department of Oncology, Johns Hopkins University School of Medicine, Baltimore, MD 21205, USA; jrubens6@jhmi.edu (J.A.R.); eraabe2@jhmi.edu (E.H.R.); 3 Neurosurgical Clinic, Medical Faculty, Heinrich-Heine University Duesseldorf, D-40225 Dusseldorf, Germany; ulf.kahlert@med.uni-duesseldorf.de; 4 Department of Neurosurgery, University of Bonn, D-53127 Bonn, Germany; Jaroslaw.Maciaczyk@ukbonn.de

**Keywords:** BCOR, neural stem cells, embryonal tumor, tumor model, polycomb repressive complex, BCORL1

## Abstract

Central nervous system tumor with BCL6-corepressor internal tandem duplication (CNS-BCOR ITD) is a malignant entity characterized by recurrent alterations in exon 15 encoding the essential binding domain for the polycomb repressive complex (PRC). In contrast to deletion or truncating mutations seen in other tumors, BCOR expression is upregulated in CNS-BCOR ITD, and a distinct oncogenic mechanism has been suggested. However, the effects of this change on the biology of neuroepithelial cells is poorly understood. In this study, we introduced either wildtype BCOR or BCOR-ITD into human and murine neural stem cells and analyzed them with quantitative RT-PCR and RNA-sequencing, as well as growth, clonogenicity, and invasion assays. In human cells, BCOR-ITD promoted derepression of PRC2-target genes compared to wildtype BCOR. A similar effect was found in clinical specimens from previous studies. However, no growth advantage was seen in the human neural stem cells expressing BCOR-ITD, and long-term models could not be established. In the murine cells, both wildtype BCOR and BCOR-ITD overexpression affected cellular differentiation and histone methylation, but only BCOR-ITD increased cellular growth, invasion, and migration. BCOR-ITD overexpression drives transcriptional changes, possibly due to altered PRC function, and contributes to the oncogenic transformation of neural precursors.

## 1. Introduction

Central nervous system (CNS) tumor with BCL6-corepressor internal tandem duplication (CNS-BCOR ITD) is a malignant entity initially identified by Sturm et al. [1], and more recently proposed as a distinct CNS tumor type by the Consortium to Inform Molecular and Practical Approaches to CNS Tumor Taxonomy (cIMPACT) [2,3]. Similar to most other new CNS tumor types in the classification, it is characterized by a common molecular alteration and shows relatively homogenous clinicopathologic features: (1) the vast majority arise in the first six years of life (Table 1, Supplementary Table S1) [1,4,5,6,7,8,9,10,11,12,13,14,15,16], as do tumors occurring in the kidney and soft tissues harboring the same BCOR-ITD alterations [17,18]; (2) mixed neuroepithelial and mesenchymal histological features can be present [2,8]; (3) additional genetic alterations are generally not found [1,9]; and (4) the recurrent BCOR-ITD alteration has been suggested as the critical oncogenic driver.

**Figure 1:**
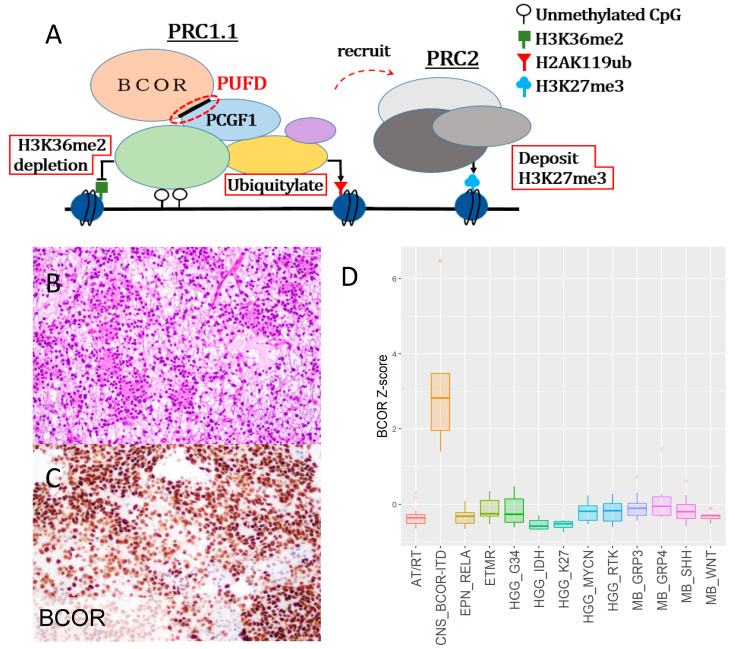
CNS-BCOR ITD is an aggressive tumor with mutant-BCOR overexpression. (**A**) Working model for epigenetic modulation of transcription by the BCOR-PRC1.1 complex, integrating published data on the PRC1.1 and PRC2 complexes. (**B**) A typical histologic appearance of CNS-BCOR ITD. (**C**). Neoplastic cells are diffusely and strongly positive for BCOR. (**D**) BCOR mRNA levels in a range of primary CNS tumor types from public data, including AT/RT (atypical teratoid rhabdoid tumor), EPN_RELA (RELA-fusion positive ependymoma), ETMR (embryonal tumor with multilayered rosettes), HGG_G34 (high-grade glioma with H3 G34 mutation), HGG_IDH (high-grade glioma with IDH1/2 mutation), HGG_K27 (high-grade glioma with H3 K27M mutation, HGG_MYCN (high-grade glioma with MYCN amplification), HGG_RTK (high-grade glioma driven by receptor tyrosine kinases), MB_GRP3 (group 3 medulloblastoma), MB_GRP4 (group 4 medulloblastoma), MB_SHH (sonic-hedgehog-activated medulloblastoma), MB_WNT (WNT-activated medulloblastoma). We confirmed significant upregulation in CNS-BCOR ITD (nine samples) compared to other CNS tumor types (12 entities, 131 samples) (*p* < 0.0001, Mann–Whitney test).

BCOR was originally identified as an interacting corepressor of B-cell lymphoma 6 protein (BCL6), and recent studies have revealed a genome-wide transcriptional impact via interactions with polycomb repressive complex 1.1 (PRC 1.1) [19]. BCOR-PRC 1.1 modifies the epigenome in three different ways: it demethylates H3K36me2 into H3K36me1, monoubiquitylates H2AK119, and recruits PRC2, which deposits H3K27me3 repressive chromatin marks (Figure 1a) [20]. In studies performed using murine embryonic stem cells, BCOR was shown to be necessary for the formation of primitive erythrocytes, for B- and T-cell development, and for the timely expression of genes regulating pluripotency and ectodermal and mesodermal development [21]. In humans, germline loss of *BCOR* induces oculo-facio-cardio-dental syndrome (OFCD) [22], which is inherited in an X-linked dominant mode and is lethal in males.

The BCOR-ITD alteration occurs within a domain needed for binding to PCGF proteins known as the PCGF Ub-like fold discriminator (PUFD), which is essential for functioning of the PRC1.1 complex as an epigenetic modifier [19]. This suggests aberrant epigenetic activities as a possible mechanism of tumorigenesis in tumors with BCOR-ITD. In contrast to the BCOR deletion or truncating mutations frequently seen in other tumors such as medulloblastoma [23,24] and myeloid malignancies [25], BCOR levels are upregulated in CNS tumors with BCOR-ITD alterations [1]. While BCOR-ITD is thought to have an oncogenic mechanism distinct from mutation or deletions in the locus, it is not yet clear if the ITD causes loss-of-function, gain-of-function, or neomorphic effects.

In a study comparing gene expression in clear cell sarcoma of the kidney (CCSK) harboring BCOR-ITD to Wilms tumors, upregulation of PRC2 targets were described, suggesting disruption of polycomb regulation as a possible pathogenic mechanism [18]. However, PRC upregulation was not highlighted in the original study of CNS tumors with BCOR alterations when they compared expression within four newly described tumor entities [1].

Few models exist in which the oncogenic functions of BCOR-ITD can be examined, or potential therapies tested. One patient-derived cell line with CNS-BCOR ITD has been reported and showed upregulation of insulin growth factor 1 receptor (IGF1R) and insulin growth factor 2 (IGF2) [7]. To investigate the role(s) of wildtype BCOR (BCOR-WT) or BCOR-ITD in controlled systems relevant to brain tumors, we introduced them into human and murine neural stem cells (hNSC and mNSC) by lentiviral transduction. These isogenic lines were used to examine the effects on transcription, differentiation, and cellular activities, providing new insights into the potential oncogenic roles of BCOR-ITD.

## 2. Results

### 2.1. CNS-BCOR ITD is an Aggressive Tumor with Overexpression of Mutant-BCOR

BCOR overexpression has been described at both the mRNA and protein levels in CNS-BCOR ITD and is generally diffuse. We show a molecularly confirmed case similar to those in previous studies, with scattered poorly formed rosettes and myxoid changes (Figure 1b) as well as strong, diffuse nuclear BCOR protein expression (Figure 1c). BCOR mRNA expression in primary CNS tumors was evaluated using public data, and we confirmed the upregulation in CNS-BCOR ITD (nine samples) compared to all other CNS tumor types (12 entities, 131 samples) (Figure 1d, *p* value < 0.0001, Mann–Whitney test). The median overall survival time for CNS-BCOR ITD calculated by the Kaplan–Meier method was three years, and this prognosis was inferior to that of all major medulloblastoma (MB) groups except group 3 (Supplementary Figure S1, *p* value < 0.0001 vs. WNT activated MB, *p* value = 0.0038 vs. sonic-hedgehog-activated MB, *p* value = 0.0031 vs. group 4 MB, Log-rank test). Of the 27 cases with more detailed clinical information available, metastasis outside the CNS was observed in four cases, which is highly unusual in CNS tumors [26] (Supplementary Table S1).

### 2.2. CNS BCOR-ITD Shows Upregulation of PRC2 Targets, and hNSC with BCOR-ITD Recapitulated This Induction

**Figure 2:**
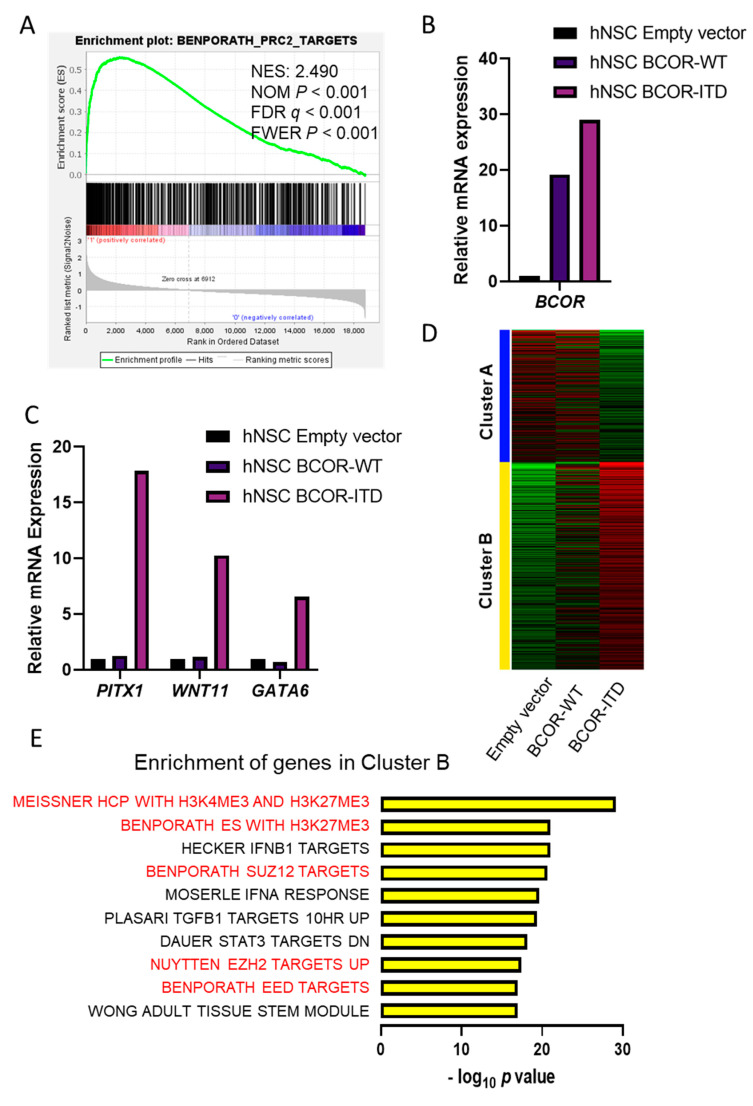
BCOR-ITD is associated with upregulation of PRC2 targets in primary tumors and neural stem cells. (**A**) GSEA of CNS-BCOR ITD primary tumors revealed significant upregulation of PRC2 targets. (**B**) Increased expression of BCOR mRNA in BCOR-WT and BCOR-ITD hNSC models. (**C**) Increased mRNA expression of PRC2-target genes PITX1, WNT11, and GATA6 in hNSC with BCOR-ITD determined by RTqPCR. (**D**) K-means clustering identified two clusters of differentially expressed genes between hNSC with empty vector, BCOR-WT, and BCOR-ITD. (**E**) Top 10 gene sets from MsigDB c2 enriched in Cluster B, with PRC2 or H3K27me3 related gene sets highlighted in red.

An expression matrix of mRNAs in CNS-BCOR ITD (*n* = 9) and other primary CNS tumors (*n* = 163) was utilized for gene-set enrichment analysis (GSEA) using MSigDB-curated gene sets. Of the top 10 most significantly enriched gene sets identified, seven were related to PRC2-targets or associated with the H3K27me3 mark (Figure 2a and Supplementary Table S2) (GSEA with family-wise error rate (FWER), *p* value ≤ 0.01). Significant enrichment of the hedgehog signaling pathway (GSEA with FWER, *p* value = 0.048) was also found in CNS-BCOR ITD, consistent with the previous study [1]. Introduction of a full length BCOR-ITD constructed into hNSC resulted in increased mRNA expression of the mutant form as compared to native BCOR-WT (Figure 2b). These BCOR-ITD transduced cells also showed a greatly induced expression of three PRC2-targets examined by reverse transcription quantitative polymerase chain reaction (RTqPCR): PITX1, WNT11, and GATA6. No such increase was seen in hNSC transduced in parallel with empty vector or BCOR-WT (Figure 2c), nor in HEK293T cells transduced with BCOR-ITD (Supplementary Figure S2A).

This model was also analyzed by RNA sequencing (RNA-seq), and we performed k-means clustering to identify genes upregulated in hNSC with BCOR-ITD (Figure 2d). Examination of differentially upregulated genes (*n* = 1259) revealed that five of the top 10 most significantly enriched gene sets were PRC2 targets, recapitulating important aspects of the signature of primary CNS-BCOR ITD tumors (Figure 2e and Supplementary Table S4) (adjusted *p* value < 1.13 × 10^−17^).

Contrary to our expectation, hNSC BCOR-ITD with upregulated PRC2 targets showed no growth advantage, and the expression levels of mutant BCOR decreased by over 10 to 30 passages (data not shown). Therefore, we could not establish a long-term BCOR-ITD model in these human-derived cells.

### 2.3. Both BCOR^E7–15^-WT and BCOR^E7–15^-ITD Alter Differentiation of mNSC

**Figure 3:**
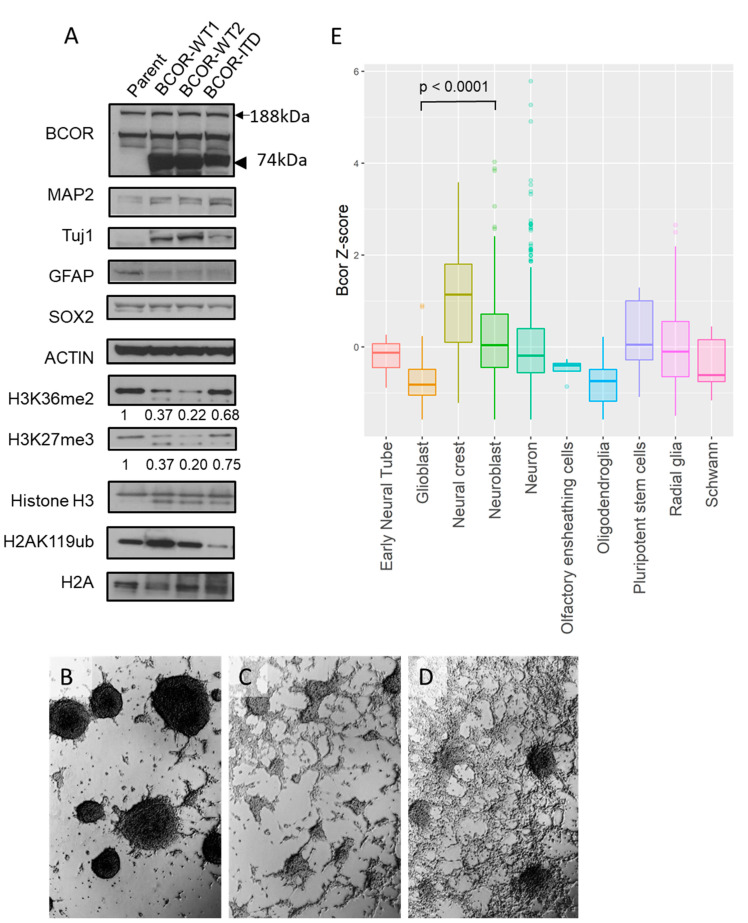
Both BCOR^E7–15^-WT and BCOR^E7–15^-ITD alter differentiation of mNSC. (**A**) Representative western blots of BCOR, MAP2, Tuj1, GFAP, SOX2, beta-Actin, H3K36me2, H3K27me3, histone H3, H2AK119Ub, and histone H2A in mNSC, mNSC BCOR^E7–15^-wildtype (WT1 and WT2), and mNSC BCOR^E7–15^-ITD. Arrow and arrowhead indicate endogenous and exogenous BCOR, respectively. (**B**–**D**) Microscopic images of parent mNSC cells (**B**), mNSC BCOR^E7–15^-WT (**C**), and mNSC BCOR^E7–15^-ITD (**D**). (**E**) Bcor mRNA expression in mouse developing brains single cell RNA-seq. A significant difference between glioblast to neuroblast (*p* < 0.0001, Mann–Whitney test) is indicated.

We next examined the effects of BCOR expression in NSC isolated from mice, but BCOR-ITD protein expression levels were much lower than isogenic mNSC transduced in parallel with BCOR-WT. As a high-level expression of BCOR-ITD is a key feature of primary human tumors, we sought to generate a model with enhanced expression rather than examine these cells further. To increase BCOR expression, we generated shortened constructs containing only the essential PUFD, Ankyrin (ANK) repeats and MLLT3 binding domains (BCOR^E7–15^; BCOR exons 7 to 15) with or without the ITD. After transfecting these into mNSC, we were able to confirm robust protein expression of the ITD form by western blot (Figure 3a, top panel, 74 kDa) as compared to endogenous protein (188 kDa).

The cellular morphology of the mNSC changed after the introduction of the BCOR constructs. While the original parental cells largely grew as neurospheres (Figure 3b), increased BCOR-WT expression resulted in more adherent growth (Figure 3c), and this was further accentuated in the mNSC BCOR-ITD cultures (Figure 3d). These changes suggest that the BCOR constructs could be promoting cellular differentiation. This possibility was examined by western blot utilizing neuronal, glial, and stem cell markers (Figure 3a). Both BCOR^E7–15^-WT and BCOR^E7–15^-ITD increased the expression of the neuronal differentiation markers MAP2 and Tuj1 but decreased the levels of the glial marker GFAP. The stem/progenitor cell markers SOX2 and Nestin were relatively stable after introduction of BCOR^E7–15^-WT and BCOR^E7–15^-ITD, as was OLIG2, while mRNA for the astrocytic marker GLAST decreased slightly (Figure 3a, Supplementary Figure S3).

To assess possible epigenetic changes, histone methylations H3K36me2, H3K27me3, and the ubiquitination H2AK119ub were also evaluated by western blot. A decrease of both H3K36me2 and H3K27me3 were observed in BCOR^E7–15^-WT, while the effect was smaller in cells with BCOR^E7–15^-ITD. H2AK119ub was not affected by BCOR^E7–15^-WT expression but decreased with BCOR^E7–15^-ITD induction.

To further examine how BCOR levels correlate with specific cell types in the CNS, we analyzed a previously published single-cell RNA-seq database of mouse developing brains. BCOR was upregulated in pluripotent stem cells as previously reported [19], but increased BCOR expression as defined by a Z-score > 2.0 was also seen in clusters defined in the database as neural crest, neuroblasts, neurons, and radial glia. Consistent with our western blot findings suggesting BCOR promotes neuronal rather than glial differentiation, neuroblasts showed significantly higher BCOR expression than glioblasts (Figure 3e and Supplementary Table S5) (*p* value < 0.0001, Mann–Whitney test).

### 2.4. BCOR^E7–15^-ITD Overexpression Increased Cellular Growth, Invasion, and Migration of mNSC, while BCOR^E7–15^-WT Decreased Cellular Growth

**Figure 4:**
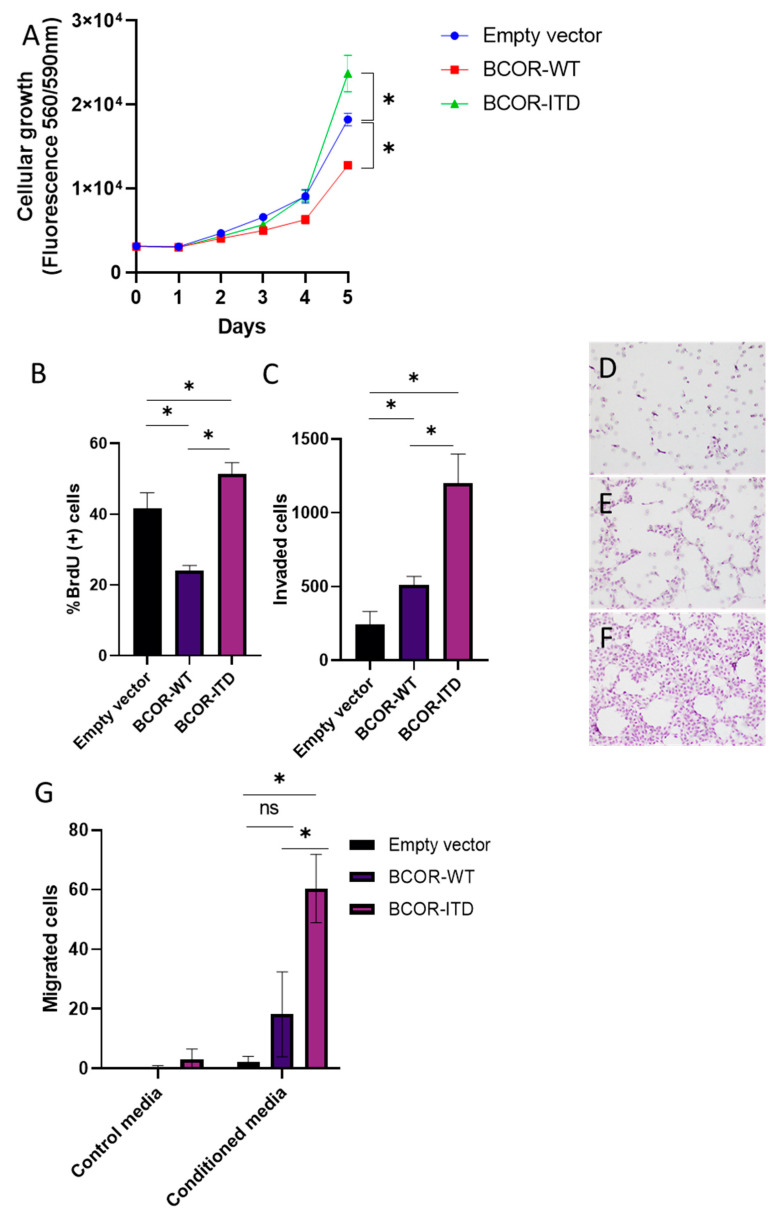
BCOR^E7–15^-ITD overexpression increased cellular growth, invasion, and migration of murine neural stem cells. (**A**) Increased growth of BCOR^E7–15^-ITD as compared to BCOR^E7–15^-WT and control mNSC with empty vector measured by cell titer blue assay. (**B**) Increased proliferation of BCOR^E7–15^-ITD as compared to BCOR^E7–15^-WT measured by BrdU incorporation. (**C**) Increased invasion of BCOR^E7–15^-ITD as compared to BCOR^E7–15^-WT and control mNSC. (**D**–**F**) Representative images of transwell invasion assay. Empty vector (**D**), BCOR^E7–15^-WT (**E**), and BCOR^E7–15^-ITD (**F**). (**G**) Increased invasion of BCOR^E7–15^-ITD as compared to BCOR^E7–15^-WT and control mNSC. (* *p* < 0.05, analyzed by Tukey’s test. Data shown as mean ± SD).

The effect of BCOR induction on cellular growth and invasion was evaluated using the mNSC BCOR^E7–15^ models. BCOR^E7–15^-ITD promoted growth as measured by cell titer blue (*p* value = 0.0008 at day five, Tukey’s test) (Figure 4a) as well as proliferation as measured by BrdU incorporation (*p* value = 0.024, BrdU, Tukey’s test) (Figure 4b). In contrast, overexpression of BCOR^E7–15^ -WT inhibited both of these as compared to mNSC with the empty vector (growth, *p* value < 0.0001 at day five, Tukey’s test) (proliferation, *p* value = 0.0013, BrdU, Tukey’s test) (Figure 4a,b).

Transwell assays were performed to evaluate invasion and migration, using as combinations of barrier and chemo-attractant matrigel/growth factor or gelatin/conditioned media, respectively. BCOR^E7–15^-ITD significantly increased these activities as compared to the empty vector control (*p* value < 0.0001, Tukey’s test) and BCOR^E7–15^-WT (*p* value ≤ 0.0001, Tukey’s test) (Figure 4c–g). Clonogenicity was evaluated with soft-agar colony formation assay, but no increase was seen in mNSC with BCOR^E7–15^-WT or BCOR^E7–15^-ITD (data not shown).

## 3. Discussion and Conclusions

In this study, we examined the effects of introducing BCOR-WT and BCOR-ITD into murine and human neural cells. While the loss of BCOR has been shown to affect normal and oncogenic development in myeloid [25], mesenchymal [27], and neural precursors [23,24], the effect of wildtype- or mutant-BCOR overexpression is more poorly understood. We found that BCOR-ITD expression results in derepression of PRC2 target genes in CNS tumors. The changes in PRC2 targets were identified using expression data from human CNS-BCOR ITD brain tumor samples, and the introduction of BCOR-ITD into hNSCs recapitulated this molecular feature. It was previously known that CCSK, a tumor arising in the kidney that frequently harbors BCOR-ITD mutation, shows PRC2 target gene derepression, but the direct effect of BCOR-ITD on transcription in genetically defined functional models had not been investigated [17,18].

Interestingly, GSEA of data from both clinical CNS-BCOR ITD and CCSK samples revealed enrichment of very similar gene sets [18], suggesting that the effects of BCOR-ITD can be similar in different tissues. Among the top 10 most enriched gene sets, most were PRC2 target genes or loci with the H3K27me3 mark in their promoters in embryonic stem (ES) cells or neural precursor cells. GSEA also showed that PRC2 target gene sets in human fibroblasts, hepatocellular carcinomas, and prostate cancers are not enriched in CNS tumors with BCOR-ITD (Supplementary Table S2), suggesting a degree of tissue specificity with respect to the effects of this oncogenic driver. In addition, the BCOR-ITD introduction into HEK293T cells did not upregulate any of three PRC2-target genes assessed (Supplementary Figure S2B), providing further support for the concept that its effects can be dependent on the cellular context. It will be interesting to further investigate if CNS-BCOR ITD and CCSK share common cellular phenotypes or pathway activation.

High-level BCOR-ITD expression could not be sustained for more than 10 to 15 passages in hNSC, and we could not achieve robust BCOR-ITD levels for even short periods on mNSC. Rapid downregulation of BCL6/ BCOR levels after initial overexpression has previously been reported [28]. The introduction of a truncated version of BCOR (BCOR^E7–15^) into mNSC, both with and without the ITD, did result in high protein levels and were used for functional studies in murine cells.

Both wildtype and ITD-containing BCOR^E7–15^ overexpression induced the neuronal differentiation of mNSC, in line with our analysis of data from a prior single-cell RNA-sequence examination of developing mouse brains showing significant BCOR upregulation in neuronal precursors compared to glial ones. This result is also at least partially compatible with a previous study investigating BCOR mRNA in murine embryos by whole mount in situ hybridization, which showed significant expression in the neural tube and dorsal root ganglion in early fetal stages and in the trigeminal ganglion, neopallial cortex, and corpus striatum in later fetal stages [29].

Although the effect on differentiation was similar after introduction of BCOR^E7–15^ -WT and BCOR^E7–15^ -ITD, changes in the proliferation, invasion, and migration of cells were quite distinct. BCOR^E7–15^ ITD significantly increased invasion and migration, while the effect of BCOR^E7–15^ -WT was much smaller. In addition, only the ITD form promoted culture growth, while BCOR^E7–15^ -WT decreased cellular growth compared to empty vector controls. In a previous study, both wildtype- and ITD-harboring BCOR overexpression in 293T increased clonogenic activity [17], but we did not observe increased clonogenicity in either our hNSC or mNSC models.

While the truncated BCOR^E7–15^ -WT and BCOR^E7–15^ -ITD generated new insights into the potential effects of this internal tandem duplication in neural cells, it will be important to continue attempting to extend these studies to full-length BCOR constructs in order to rule out any truncation-specific effects. It would also be interesting to see how BCOR^E7–15^ –ITD affects human cells. Another potential weakness with our approach is the use of a human BCOR construct for functional studies in mNSCs. However, the overall amino acid homology between mouse and human BCOR is relatively high (88.4%), as assessed by NCBI BLAST search (https://blast.ncbi.nlm.nih.gov/Blast, last accessed on 17 March 2021), and in exon 15 where the ITD resides 90.6% of amino acid sequences are identical. In addition, our BCOR^E7–15^ construct was not similar to any other proteins in mice (less than 52.1%). Nevertheless, additional studies using murine-derived constructs on mNSC will be of interest.

Overall, our findings are consistent with a role for BCOR-ITD in promoting the growth and dissemination of tumors arising from CNS progenitor cells. In our review of the literature, four of 27 CNS-BCOR ITD cases were reported with extra-CNS invasion or metastases [4,5,8,10], supporting their ability to migrate and grow in other tissues. Prognosis was dismal, with only three of 33 cases reported to have survival of over 10 years. One of these less aggressive cases showed relatively low mutant-*BCOR* expression and lacked WNT activation [13], suggesting clinical heterogeneity within the entity may be associated with BCOR expression level and/or downstream transcriptional changes. While the use of BCOR^E7–15^ in mNSC models is a potential limitation, additional studies using these and other engineered lines will hopefully enable the additional examination of molecular mechanisms associated with BCOR-ITD and facilitate testing of potential therapies.

## 4. Materials and Methods

### 4.1. CNS-BCOR ITD Tumor Samples

For the representative tumor shown in Figure 1, BCOR protein expression was evaluated by immunohistochemistry (C-10, Santa Cruz Biotechnology, Inc., Dallas, TX, USA sc-514576), and BCOR-ITD in exon 15 was confirmed by sequencing, both using standard techniques in a CLIA-certified clinical laboratory. The study was approved by the Johns Hopkins Institutional Review Board.

### 4.2. Survival Analysis

Primary literature reporting either “CNS BCOR-ITD” or “CNS high-grade neuroepithelial tumor BCOR” were reviewed, and 46 cases with confirmed BCOR-ITD in exon 15 based on sequencing were identified [2] (Supplementary Table S1). Thirty-three of these cases were reported with the status at last follow-up and the length of follow-up, and we used the data to analyze the median overall survival. As a reference, the overall survival of 612 medulloblastoma patients was obtained from a previous study [30] and analyzed at the same time (Table 1, Supplementary Figure S1).

### 4.3. BCOR Expression in Primary CNS Tumors

BCOR expression in CNS BCOR-ITD was evaluated using Affymetrix Human Genome U133 Plus 2.0 RNA-microarray expression data from a previous study [1]. Normalized expression values of 20,056 genes from 177 primary CNS tumors were obtained from the database [31] (accession number: E-GEOD-73038) and uploaded to R environment (4.0.3) for downstream analysis. From these 177 samples, we extracted nine cases with BCOR-ITD confirmed either by sequencing or PCR [2], and compared BCOR expression to other CNS malignancies: atypical teratoid rhabdoid tumors (AT/RT), RELA-fusion positive ependymoma (EPN_RELA), embryonal tumor with multilayered rosettes (ETMR), high-grade glioma with H3 G34 mutations (HGG_G34), high-grade glioma with IDH1/2 mutations (HGG_IDH), high-grade glioma with H3 K27M mutations (HGG_K27), high-grade glioma with MYCN amplification (HGG_MYCN), high-grade glioma driven by receptor tyrosine kinases (HGG_RTK), group 3 medulloblastoma (MB_GRP3), group 4 medulloblastoma (MB_GRP4), sonic-hedgehog activated medulloblastoma (MB_SHH), WNT-activated medulloblastoma (MB_WNT).

### 4.4. Gene-Set Enrichment Analysis (GSEA)

The GSEA was performed to investigate whether PRC2 targets were upregulated in CNS-BCOR ITD, as was previously shown in CCSK [18]. Calculations were performed with the GSEA program v. 4.1.0 [32,33]. The Broad Molecular Signatures Database (MSigDB v7.2) set c2 (curated gene sets) was used. We employed the same microarray dataset after normalization and compared the mRNA expressions between nine CNS-BCOR ITD samples and the other 164 samples from various CNS tumors listed above. For the comparison, the GSEA analysis involved 4642 gene sets and the expression of 18,738 genes. The GSEA program was run with 1000 randomized phenotypes for statistical significance estimation, and the default signal-to-noise metric between the two phenotypes was used to rank all genes. All *p* values reported by GSEA as zero represent values lower than 10^−3^ (1/1000 permutations).

### 4.5. Generation of hNSC Models

Human cerebral cortex neural stem cells were derived as described [34] in concordance with German law and Ethics Board evaluation. The study was also approved by the Johns Hopkins Institutional Review Board. In brief, cells were obtained by dissecting the cerebral cortex and cultured as neurospheres in media composed of a 70:30 mixture of Dulbecco’s modified Eagle medium (DMEM) and Ham’s F12, supplemented with 1% volume per volume (*v*/*v*) antibiotic–antimycotic, 2% (*v*/*v*) B27 supplement without retinoic acid, 5 μg/mL heparin, 20 ng/mL EGF, and 20 ng/mL FGF2 (NSC medium).

A construct carrying full-length wildtype BCOR gene (HsCD00330935) was purchased from the plasmID repository site (Harvard medical school, Boston, MA, USA), and an ITD mutation reported in the literature (c.5099_5212dup) [8] was added to the construct using NEBuilder^®^ HiFi DNA Assembly kits (NEB #E5520, New England Biolabs, Ipswich, MA, USA). BCOR-ITD and BCOR-wildtype lentiviral constructs were generated using the backbone vector pCDH-EF1α-MCS-IRES-NEO (CD533A-2, System biosciences, Palo Alto, CA, USA). Lentivirus was produced by transfecting 293T cells with VSV-G envelope plasmid, D8.9 gag/pol plasmid and the plasmid containing the gene of interest as described above using Lipofectamine 2000 (#11668500, ThermoFisher Scientific, Waltham, MA, USA) per the manufacturer’s instruction.

Lentivirus carrying either BCOR-wildtype, BCOR-ITD, or the backbone vector pCDH-EF1α-MCS-IRES-NEO (empty vector control) were then used for infection. Neurospheres were accutased (#00-4555-56, ThermoFisher Scientific), and then seeded in a density at 1 million cells/mL in six wells of a 24-well plate. Cells were incubated with 4 µg/µL polybrene and the lentiviruses for 24 h and then selected by G418 Sulfate (30-234-CI, CORNING, Corning, NY, USA) for 10 days. Expressions were confirmed by RTqPCR.

### 4.6. Quantitative RT-PCR (RTqPCR)

Total RNA was extracted using RNeasy Mini Kit (#74104, Qiagen, Germantown, MD, USA) with DNAse 1 digestion, and cDNAs were synthesized using a High-Capacity RNA-to-cDNA™ Kit (#4387406, Applied Biosystems, Waltham, MA, USA). After the reverse transcription, mRNA expression was evaluated by quantitative PCR using PowerUP SYBR Green Master Mix (A25742, Applied Biosystem) and primers listed in the supplementary file (Supplementary Table S3). Relative expressions compared to the empty vector control were calculated by ΔΔCt method using beta-actin (*ACTB* or *Actb* gene) as an endogenous control. Melting curves were simultaneously evaluated in all experiments to confirm a single distinct peak.

### 4.7. RNA Sequencing of hNSC Models

The cells were cultured for 10 passages under G418 selection prior to RNA sequencing. Total RNA was extracted with the same method as described above, and sequenced using standard techniques (Novogene Bioinformatics Institute, Beijing, China) after being assessed for quality and integrity using a Bioanalyzer (Agilent 2100). All samples had an RNA Integrity Number score of higher than 8.5.

For library constructions, mature mRNAs, processed and equipped with a poly(A) tail at the 3′ end, were captured via oligo(dT) beads and then fragmented randomly. The first-strand cDNA was synthesized using random hexamer primers and M-MuLV Reverse Transcriptase, and the second strand was subsequently generated by dNTPs, DNA polymerase I and RNase H. Double-stranded cDNA molecules were purified by AMPure XP beads (Beckman Coulter, Beverly, USA) and the overhanging ends were repaired to blunt ends by exonuclease/polymerase. After 5′ phosphorylation and 3′ adenylation, the cDNAs were ligated with P5/P7 sequencing adapters to prepare for hybridization and then purified with AMPure XP system (Beckman Coulter) in order to select the insert fragment of 150 to 200 bp in length. The final library was sequenced using Illumina HiSeq 4000 at pair-end 150 bps.

Sequences were mapped to the human genome GRCh37 (hg19), with more than 94% verified to have a Phred score ≥ Q30. The raw read count data was imported into a web-based platform iDEP.91 [35] (http://bioinformatics.sdstate.edu/idep/, last accessed on 11 January 2021) for downstream analysis. Of 48,162 genes, 15,651 genes with minimal counts per million (CPM) ≥ 0.5 at least in one sample were selected and transformed using EdgeR (log2 (CPM + c)). K-means clustering using the most variable 2000 genes to identify two clusters of differentially expressed genes were run, and the enrichment of genes upregulated in hNSC BCOR-ITD (cluster B in Figure 2b) was assessed using pathways in MsigDB c2 (curated) as our GSEA above. *p* values were corrected for multiple testing using false discovery rate using the iDEP.91 platform.

### 4.8. Examination Bcor Expression in Developing Brains Using Public Single-Cell RNA-seq Data

Single-cell RNA-seq data from mouse developing brain [36] and mRNA expression values of 31,053 genes aggregated per cellular “cluster” were extracted from the publicly available web page (http://mousebrain.org/downloads.html, last accessed on 8 January 2021). We calculated the Z-score of BCOR expression in each cluster and summarized it per the “class” label attributed to each cluster (Figure 2a). All 799 clusters of neuroepithelial cells with BCOR Z-score were listed in the supplementary table (Supplementary Table S5).

### 4.9. Generation of mNSC Models

Murine neural stem cells were obtained by dissecting the hindbrain of C57BL/6 mouse embryo at E12 and cultured as neurospheres. To achieve BCOR overexpression, lentiviral vectors carrying full-length BCOR genes were shortened to contain only essential PUFD, ANK repeats and MLLT3 binding domains (BCOR^E7–15^) using the Q5^®^ Site-Directed Mutagenesis kit (E0554S, New England Biolabs), then confirmed by sequencing. Lentivirus was produced and used for infection as described above. Expression was confirmed by RTqPCR and western blot.

### 4.10. Western Blotting and Antibodies

Western blots were performed by extracting cell pellets using RIPA buffer. Protein concentrations were quantified using Bradford Assay [37]. Antibodies were used according to the manufacturer’s instructions: BCOR (12107-1-AP) (from Proteintech, Rosemont, IL USA), GFAP (#3670), Histone H3 (#14269), H2AK119Ub (#8240), H2A (#12349), Nestin (#33475) (from Cell Signaling Technologies, Beverly, MA, USA), β-Actin (#47778), MAP2 (#20172) (from Santa Cruz Biotechnology), Tuj1 (MAB5564), H3K27me3 (07-449), Olig2 (AB9610) (from Sigma-Aldrich, St. Louis, MO, USA), H3K36me2 (#39255) (from Active Motif, Carlsbad, CA, USA), SOX2 (AM2048a) (from Abcepta, San Diego, CA, USA). Densitometry was performed using ImageJ v1.440 software.

### 4.11. Growth and Proliferation Assays

A CellTiter-Blue cell viability assay (G8080, Promega, Madison, WI, USA) was used to assess the growth of transduced cells. NSCs were accutased and plated at 3000 cells per well in a 96-well plate. All cellular conditions were plated in triplicate for measurements on day zero to day five. Cells were incubated with the CellTiter-Blue reagent for one hour and reduction of resazurin to resorufin was measured by recording fluorescence (560(20)Ex/590(10)Em) using a TECAN plate reader. Bromodeoxyuridine (BrdU) incorporation assay was used to determine the proliferative activity of cells as described previously [38]. Briefly, cells were incubated with 10 µM BrdU (B5002, Sigma-Aldrich) for 2 h then fixed with 70% (*v*/*v*) ethanol overnight. The DNA was denatured with 2 N HCl and the cells were permeabilized with 0.1% (*v*/*v*) Triton X-100. The cells were stained with BrdU-PE (# 12-5071-42, ThermoFisher Scientific), then analyzed with Muse Cell Analyzer (Millipore Corporation, Billerica, MA, USA).

### 4.12. Transwell Invasion and Migration Assays

The transwell invasion assay was performed as previously described [39]. Briefly, cell culture inserts (6.5 mm, Falcon insert, 8-μm pore size; Becton Dickinson, Franklin Lakes, NJ, USA) were pre-coated with Matrigel (#356231, CORNING) diluted 1:20 in serum-free DMEM/F12 medium. Cells were accutased and resuspended in serum-free DMEM/F12 medium in the upper chamber (50,000 cells in 250 μL) and 800 μL of NSC medium containing growth factors was added to the lower chamber. After a 72-h incubation, cells remaining on the upper surface of the filter were removed, and cells that had invaded through the matrigel to the lower surface were fixed with ethanol, stained with hematoxylin ((H9627, Sigma-Aldrich), and photographed. Data represent the mean of the number of cells counted in five random low-power fields (LPFs).

For transwell migration assays, NIH3T3 cells were cultured in DMEM/F12 media supplemented with 10% (*v*/*v*) FBS for 24 h, and the media was then used as conditioned media to attract cells through cell culture inserts precoated with gelatin (G1393, Sigma-Aldrich). After a 48-h incubation, cells that had migrated to the lower surface were fixed with ethanol, stained with hematoxylin (H9627, Sigma-Aldrich), and photographed. Data represent the mean of the number of cells counted in five random high-power fields (HPFs).

### 4.13. Colony Formation Assay

hNSCs and mNSCs were triturated to single cells and plated in 0.5% (*v*/*v*) soft agar (18300012, Gibco, Gaithersburg, MD, USA) in NSC media to form colonies. Upper media with growth factors was changed every three days. After 14 to 20 days, wells were stained with Crystal violet (C0775, Sigma-Aldrich), and spheres greater than 100 μm in diameter were counted.

### 4.14. Statistical Analysis

Statistical analyses were conducted with GraphPad Prism v. 8.4.2 (GraphPad Software, San Diego, CA, USA) unless otherwise specified. At least two independent experiments were performed for each cell culture assay. *p* values of < 0.05 were considered significant. For gene expression analyses and in vitro functional studies, the normality of distribution was evaluated using a Shapiro–Wilk test. Survival time was plotted using the Kaplan–Meier method, and differences in survival were analyzed by log rank analysis. BCOR expression in CNS-BCOR ITD and other primary CNS tumors was analyzed using the Mann–Whitney test. In GSEA analyses, FWER was used to determine the significance of enrichments in the context of multiple tests, and *p* values were generated as described in Section 4.4. The iDEP.91 program was used for k-means clustering analyses as specified in Section 4.7, with *p* values corrected for multiple testing using the false discovery rate. BCOR expression in the neuroblasts and glioblasts of developing mouse brains was analyzed using the Mann–Whitney test. For statistical analyses of growth, proliferation, invasion, and migration assays, in which both BCOR-ITD and BCOR-WT constructs were compared to empty vector, Tukey’s multiple comparison test was used.

## Figures and Tables

**Table 1:** Summary of molecularly confirmed cases of CNS-BCOR ITD.

	CNS-BCOR ITD
Gender	Male: Female = 1:1.05
Median Age at Diagnosis	4 years old (range: 0 to 22 years old)
Locations of Tumor (Frequency)	Cerebellum (46%), Cerebral hemisphere (44%), Other posterior fossa (7.5%), Basal ganglia (2.5%)
Median Overall Survival	3 years

## Data Availability

RNA-microarray data analyzed in this study are openly available in the refine.bio repository site (https://www.refine.bio, last accessed on 8 April 2021) under accession number E-GEOD-73038. Single-cell RNA-seq data from mouse developing brain are publicly available in the website (http://mousebrain.org/downloads.html, last accessed on 8 January 2021).

## Supplementary Materials

The following are available online at https://www.mdpi.com/article/10.3390/ijms22083913/s1, Supplementary Figure S1: Overall survival of CNS-BCOR ITD comparing to other pediatric CNS tumors. Supplementary Figure S2: Expressions of PRC2-target genes in HEK293T BCOR-ITD and mNSC BCOR^E7–15^-ITD. Supplementary Figure S3: Other differentiation markers in mNSC BCOR^E7–15^. Supplementary Table S1: Clinical features of the 46 reported cases of CNS-BCOR ITD with confirmed BCOR exon 15 internal tandem duplication. Supplementary Table S2: Summary of gene-set enrichment analysis, CNS-BCOR ITD (*n* = 9) vs Other primary CNS tumors (*n* = 163). Supplementary Table S3: List of PCR primers in this study. Supplementary Table S4: Enrichment of genes in K-means Clustering of hNSC BCOR models. Supplementary Table S5: Bcor-expressing-cells in the mouse developing brain.

## Abbreviations

BCL6	B-cell lymphoma 6 protein
BCOR-WT	Wildtype BCOR
BCOR-ITD	BCOR internal tandem duplication
BCOR^E7–15^	BCOR exons 7 to 15
CCSK	Clear cell sarcoma of the kidney
CNS	Central Nervous System
CNS-BCOR ITD	CNS tumor with BCL6-corepressor internal tandem duplication
cIMPACT	Consortium to Inform Molecular and Practical Approaches to CNS Tumor Taxonomy
FWER	Family-wise error rate
GSEA	Gene-set enrichment analysis
hNSC	Human neural stem cells
IGF1R	Insulin Growth Factor 1 Receptor
IGF2	Insulin Growth Factor 2
mNSC	Murine neural stem cells
NSC	Neural stem cells
OFCD	Oculo-facio-cardio-dental syndrome
PRC	Polycomb repressive complex
PUFD	PCGF Ub-like fold discriminator
RTqPCR	Reverse transcription quantitative polymerase chain reaction
RNA-seq	RNA sequencing
