# Supplementary material for: BCOR Internal Tandem Duplication Expression in Neural Stem Cells Promotes Growth, Invasion, and Expression of PRC2 Targets
Source: Int J Mol Sci. 2021 Apr 10;22(8):3913. doi: 10.3390/ijms22083913 (PMC8070097; doi:10.3390/ijms22083913)

Supplementary figure 1

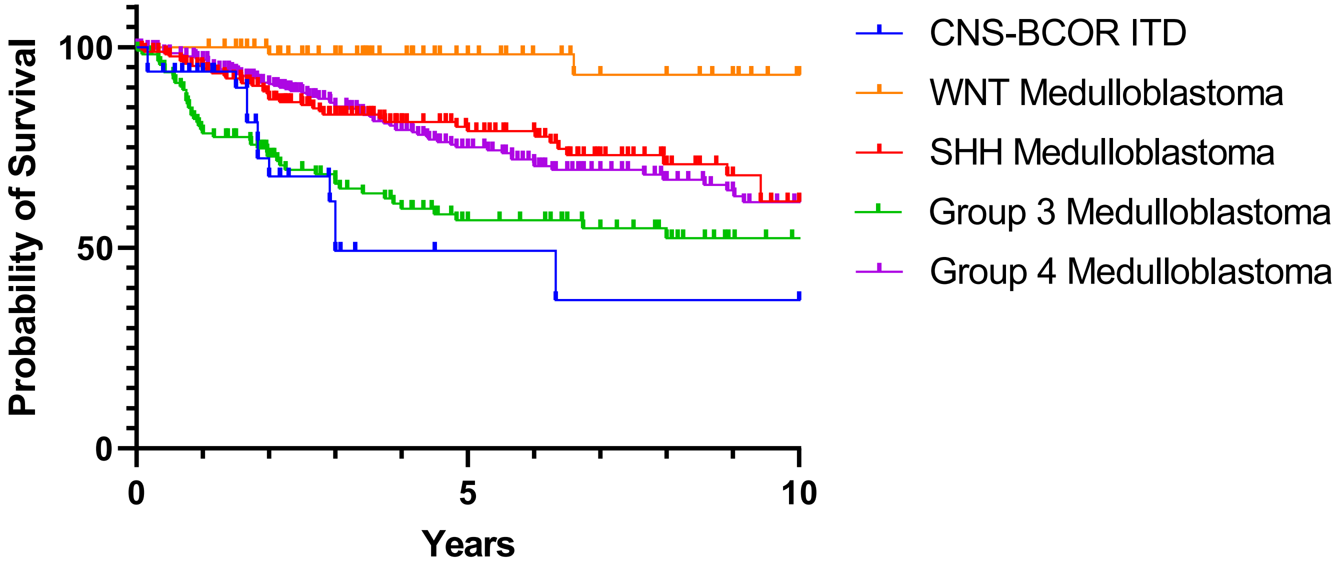

CNS-BCOR ITD

Number of cases	33
# censored subjects	21
# deaths	12
Median survival	3

Supplementary figure 2

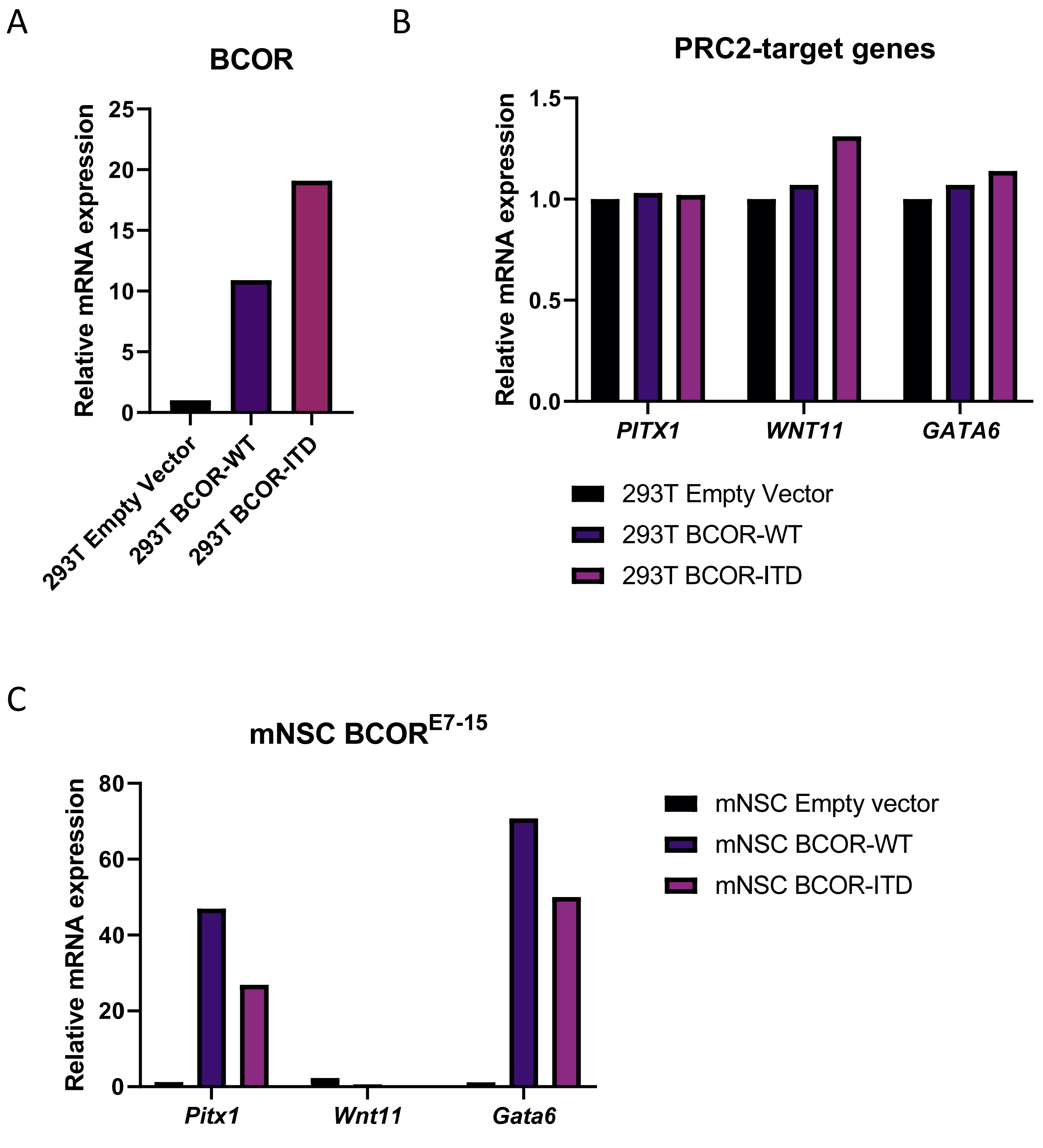

Supplementary figure 3

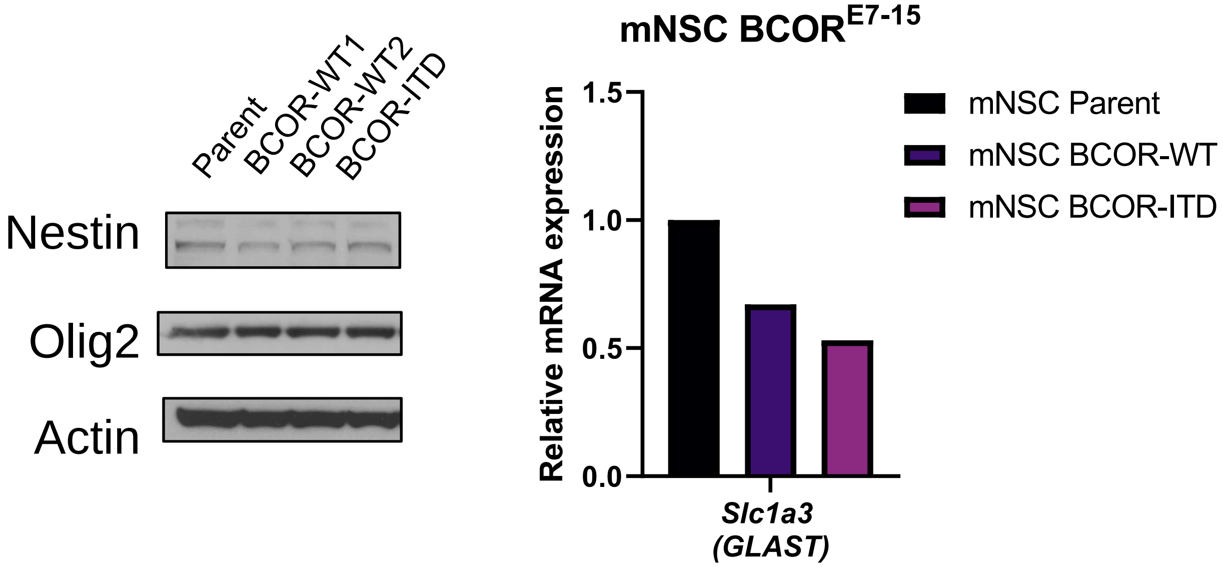

Supplement: Supplementary file 1 [file ijms-22-03913-s001.zip › ijms-1147273 - supplementary figures.pdf]
